# The Health and Physical Education Curriculum: Does It Address Muscular Fitness?

**DOI:** 10.3390/jfmk11010040

**Published:** 2026-01-18

**Authors:** Andrew Sortwell, Rodrigo Ramirez-Campillo, Urs Granacher, Christopher Joyce, Pedro Forte, Daniel A. Marinho, Ricardo Ferraz, Kevin Trimble

**Affiliations:** 1 School of Education, The University of Notre Dame Australia, Sydney 2007, Australia; 2 School of Health Sciences, The University of Notre Dame Australia, Fremantle 6160, Australia; 3 Research Centre in Sports Sciences, Health Sciences and Human Development (CIDESD), University of Beira Interior, 6201-001 Covilhã, Portugalricardo.ferraz@ubi.pt (R.F.); 4 Exercise and Rehabilitation Sciences Institute, Faculty of Rehabilitation Sciences, Universidad Andres Bello, Santiago 7591538, Chile; rodrigo.ramirez@unab.cl; 5 Sport Sciences and Human Performance Laboratories, Instituto de Alta Investigación, Universidad de Tarapacá, Arica 1010069, Chile; 6 Department of Physical Activity Sciences, Universidad de Los Lagos, Santiago 8320000, Chile; 7 Department of Sport and Sport Science, Exercise and Human Movement Science, University of Freiburg, 79102 Freiburg, Germany; urs.granacher@sport.uni-freiburg.de; 8 Department of Sports, Higher Institute of Educational Sciences of the Douro, 4560-708 Penafiel, Portugal; 9 Department of Sports Sciences, Instituto Politécnico de Bragança, 5300-253 Bragança, Portugal; 10 Research Centre for Active Living and Wellbeing (Livewell), Instituto Politécnico de Bragança, 5300-253 Bragança, Portugal; 11 Department of Sports Sciences, University of Beira Interior, 6201-001 Covilhã, Portugal

**Keywords:** muscle strength, resistance training, child health, adolescent health, physical education

## Abstract

**Background**: The World Health Organization and the Australian physical activity guidelines, in line with contemporary research, recommend regular muscle-strengthening activities for optimal muscular fitness in children and adolescents. However, the extent to which muscle-strengthening or muscular fitness receives curricular emphasis is unknown in Australia. **Objectives**: To examine to what extent the Australian Health and Physical Education Curriculum, Foundation to Year 10 (AHPEC; F–10) addresses and/or promotes muscular fitness. **Methods**: This study involved a mixed-methods content analysis of the AHPEC F–10 using: (i) conceptual analysis to identify muscular fitness-related terms; and (ii) relational analysis to examine alignment between muscular fitness content and curriculum rationale/aims. A search of national and international physical activity guidelines and school-based muscular fitness intervention literature generated a keyword set to guide abstraction from the AHPEC. Curriculum aim, rationale, level descriptions, achievement standards and content were coded to determine the extent to which muscular fitness was embedded. Intercoder reliability was established via consensus meetings. Muscular fitness content coverage was quantified as the proportion of directly aligned muscular fitness relevant content points per stage and aggregated primary (F–6), secondary (7–10), and F–10 scores. **Results**: A review of 32 national and one international physical activity guidelines identified 88 muscular fitness activities in total, with some activities appearing in multiple guidelines; 53.1% of national guidelines did not provide explicit muscular fitness examples, and where examples existed, they emphasised accessible modes (e.g., climbing, bodyweight tasks, jumping, and lifting). Additionally, analysis of school-based muscular fitness intervention literature identified 22 distinct muscular fitness activities to guide abstraction. Muscular fitness was absent in the AHPEC rationale and aims, was largely inferred in primary years level description and achievement standards and became more explicit in secondary achievement standards. Direct alignment of content with muscular fitness was non-existent or low across stages of learning (Foundation = 0%, Stage 1 = 0%, Stage 2 = 6.1%, Stage 3 = 9.1%, Stage 4 = 8.6%, Stage 5 = 8.8%). Overall, muscular fitness content coverage averaged 3.8% in primary, 8.7% in secondary, and 5.4% across F–10. **Conclusions**: The AHPEC treats muscular fitness as a low priority in primary schooling and a minor content area in secondary, yielding developmental messaging that is less aligned with contemporary evidence and physical activity guidelines.

## 1. Introduction

In 2020 the World Health Organization (WHO) updated its physical activity guidelines, recommending that children and adolescents aged 5–17 years engage in ≥60 min of moderate-to-vigorous physical activity (MVPA) daily, along with vigorous aerobic activities and muscle- and bone-strengthening exercises at least three times per week [1]. However, <20.0% of females and males aged 11–17 years comply with the WHO recommendations for physical activity. Moreover, females are more likely to be inactive than males (85.0% and 77.6%, respectively) in the 11–17 years age group across most countries [2]. Across Australian jurisdictions, available data from both self-report and parent-reported surveys suggest that 19.0–67.0% of primary school-aged children and 11.0–31.0% of secondary school-aged adolescents meet the national physical activity guidelines [3].

At the core of the Australian Health and Physical Education Curriculum (AHPEC) for school children and adolescents is the acquisition of movement skills and concepts to enable students to participate in a range of physical activities confidently, competently and creatively, with the intention of supporting sustained long-term engagement in physical activity [4,5]. Despite the objectives of AHPEC, measured movement skill data show that only 36.0% of Australian females and only 41.0% of Australian males in Year 6 (i.e., Grade 6) demonstrate proficiency in locomotor movement skills, with 25.0% and 54.0%, respectively, demonstrating proficiency in object-control movement skills [6], suggesting a misalignment between curricular intent and observed developmental outcomes, and recently, there has been no improvement in physical activity engagement data for Australian children and adolescents [3]. Furthermore, as noted in an Australian longitudinal study, muscular fitness tends to track from childhood to adulthood, highlighting childhood and adolescence as critical periods for curricular emphasis [3]. Moreover, Faigenbaum et al. [7] made a strong case in a review article to prioritise muscular fitness in youth physical activity guidelines. Accordingly, this manuscript examines whether and how muscular fitness is explicitly linked within AHPEC to its intended learning outcomes and associated content expectations.

Muscular fitness is an umbrella term that includes muscle strength and power as well as local (i.e., specific) muscular endurance [8,9,10]. Low muscular fitness levels in children are associated with poor fundamental movement skills, functional limitations, and adverse health outcomes such as obesity, cardiovascular disease, and mental health disorders, all of which also accelerate biological ageing [11,12,13]. Recent evidence further indicates that muscular fitness among children and adolescents has declined over successive generations, with Australian data showing a 5.6% and 5.8% reduction in standing long jump performance among boys and girls, respectively, since the year 2000 [14,15]. Similarly, a decline in muscular strength was observed in English 10-year-olds between 1998 and 2014 [16]. These trends are consistent across various countries, including Slovenia, Spain, New Zealand, Germany and USA [17,18,19,20].

From a developmental perspective, muscular fitness training for children and adolescents reduces the risk of paediatric dynapenia, mitigates sarcopenia, elevates resting metabolic rate, reduces visceral adiposity, improves insulin sensitivity, and enhances cardiovascular and lipid profiles [21,22]. This is problematic, given emerging evidence that muscular fitness, particularly through resistance training, is a central determinant of healthy ageing and disease prevention. Moreover, large-scale epidemiological data indicate that adults engaging in regular resistance training exhibit significantly longer telomeres, a biomarker of cellular ageing, than non-participants, equivalent to nearly four years less biological ageing with only 90 min of weekly training [23]. These adaptations collectively counteract the progression of chronic diseases such as obesity, diabetes, and cardiovascular disorders, all of which accelerate biological ageing and thus, muscular fitness serves not only as a performance-oriented pursuit but as a potent biological intervention with profound implications for lifelong health span and functional independence throughout life [24,25,26]. Hence, lifelong engagement in muscular fitness activities should be fostered in early life.

Despite the growing evidence supporting the benefits of muscle- and bone-strengthening exercises for school-aged children [27,28,29,30], the emphasis on accumulating 60 min of MVPA daily often overshadows the importance of enhancing muscular fitness early in life [28,31]. However, Health Physical Education (HPE) school programmes in Australia and internationally offer a unique platform to prioritise muscular fitness development alongside MVPA by incorporating targeted muscular fitness or strength exercises into the curriculum, which might not otherwise be available to children [32,33].

Public health professionals and paediatric researchers recognise schools as strategic settings for promoting physical activity as a lifelong habit [1,34,35]. Most 5 to 7-year-old children already possess the emotional maturity to engage in sports activities, and they may also be prepared for systematic muscle and bone-building exercises tailored to their needs, talents, and interests [29], as recommended by WHO and the aligned Australian Physical Activity Guidelines [36]. The key learning area in schools, HPE, has the potential to provide age-appropriate muscular fitness activities that help build the foundation needed for neuromuscular motor performance and ongoing physical activity.

Integral to the key learning area of HPE is students’ acquisition and application of movement skills, concepts and strategies across a range of physical activity contexts, which includes exercise [5]. The most effective exercise interventions often involve muscular fitness or resistance training activities such as free weights, body weight exercises, weight machines, neuromuscular integrative training and plyometric activities [28,37]. Therefore, muscular fitness and resistance training represent an important physical activity context for HPE [38,39]. Indeed, AHPEC in schools offers an appropriate setting for delivering timely and comprehensive education on muscular fitness within the physical activity context. The Australian Government Department of Health, National Preventive Health Strategy 2021–2030, highlights the importance of creating educational programmes and resources for schools to support physical development and wellbeing [40]. Yet, it is unclear if muscular fitness education is specifically mandated in the Australian school curriculum and, to date, no research has investigated muscular fitness content in the F–10 AHPEC.

The overarching aim of this study was to examine the role of muscular fitness within international physical activity guidelines and to analyse whether it holds a comparable role in the AHPEC. Collectively, the study focuses on policy prescription, curriculum coverage, alignment with stated aims, and opportunities for strengthened integration of muscular fitness across schooling. Specifically, the study evaluated the extent to which the F–10 AHPEC addresses students’ muscular fitness needs, with particular attention to the provisions and guidance in the F–6 and 7–10 curricula. Therefore, this study aimed to address the following five research questions: (1) To what extent do national and international physical activity guidelines for school-aged children and adolescents prescribe muscular fitness, and which specific activities are identified as contributing to muscular fitness development? (2) To what extent does the F–6 AHPEC address the muscular fitness needs of students? (3) To what extent does the 7–10 AHPEC address the muscular fitness needs of students? (4) Does the AHPEC rationale and aims align with an adequate inclusion of muscular fitness content? and (5) Where could muscular fitness content be best integrated? To address these questions, we conducted a content analysis of the relevant curriculum documents and guideline statements. Content analysis can be broadly defined as a systematic, replicable procedure for compressing large amounts of textual data into fewer content categories based on explicit coding rules [41]. It is a qualitative research method frequently used to analyse textual information in a standardised way that allows evaluators to draw inferences about the meaning and emphasis of that information [42].

## 2. Materials and Methods

### 2.1. Methodological Foundations

The study employed a mixed-methods analytic approach to determine the extent to which muscular fitness exists in the AHPEC Version 9.0 and to identify underlying themes and patterns that are not readily discernible through direct observation. The analysis incorporated quantitative (i.e., frequency-based) factor analysis and qualitative interpretation. The framework used for conducting the content analysis was guided by three key elements: objectivity (i.e., maintaining rigorous research standards), system (i.e., conducting a systematic content analysis), and generality (i.e., the relevance of the study’s findings) [43]. To establish objectivity, each step in the research process was carried out according to specific pre-determined rules and procedures that were first piloted [43]. The principle of generality was incorporated into the study by evaluating the content’s relevance and systematically comparing the text analysis results with other document attributes. Attributes included the curriculum and the Level of Description statements for each stage of learning, the aims of the HPE curriculum, recent research, Australian and WHO physical activity guidelines, and the intended student setting (i.e., primary, secondary) in which the curriculum is delivered [44]. By situating the analysis within a broader contextual framework, the study sought to ensure a more comprehensive understanding of the extent to which muscular fitness is embedded in the curriculum, thereby enhancing the interpretability, depth and robustness of the findings.

The principle of system was operationalised through a staged and replicable analytical process. In conducting the content analysis, the categories and coding rules were pre-determined before data collection and applied consistently throughout the study [44]. This approach combined systematic frequency counts with qualitative interpretation of curricular meaning and emphasis. This systematic approach to reading the curriculum text distinguished the analysis from more general forms of interpretation, as the pre-determined coding framework narrowed the focus and enabled the identification of trends, patterns, and relationships in the data [41,45]. Furthermore, the process was employed to minimise researcher bias and enhance the accuracy of the analysis. To ensure that these coding rules and guidelines were consistently applied, measures to establish trustworthiness were put in place. For this study, these measures included having experts in the fields of Strength and Conditioning (A.S., R.R.C., U.G., P.F., R.F., C.J., D.M.), Australian curriculum policy (K.T., A.S.), HPE (R.F., K.T., A.S.), and HPE curriculum development (A.S., K.T., R.F.) to improve and develop the systematic approach. Furthermore, the sample data sets (i.e., three stages of learning) were analysed by three experts using the pre-established rules and procedures separately (see Section 2.4). The results from the analysis of the sample data sets were then compared to assess the feasibility of the piloted coding procedures before commencing the initial stage of the content analysis for the current study. In so doing, the trustworthiness of the content analysis procedures was established, the coding framework was developed and implemented (P.F., K.T.), and intercoder reliability was subsequently confirmed [46] through agreement with an HPE curriculum-writing expert (A.S.), with no discrepancies identified.

### 2.2. Conceptual and Relational Content Analysis

The study followed three phases of data analysis as described by Elo and Kyngäs [47]: (i) preparation of the unit of analysis and data collection; (ii) organisation of coding and abstraction; and (iii) reporting synthesis of results. A combination of conceptual and relational content analysis was employed to evaluate the extent to which the AHPEC addressed and promoted the development of students’ muscular fitness needs. Conceptual content analysis, which identifies the existence and frequency of concepts in text [48], was used to systematically categorise curriculum content related to muscular fitness and quantify the occurrence of relevant words, concepts, or meanings.

Relational content analysis was employed to qualitatively examine how the muscular fitness concepts were positioned and connected within the curriculum textual data [49]. Building on conceptual analysis, this approach explored how muscular fitness was positioned in relation to broader curriculum aims and rationale, and whether these high-level statements were coherently reflected and or linked to specific opportunities for muscular fitness development within the content [50]. In line with the principle of generality [44], the analysis therefore assessed the degree of alignment across the curriculum layers, evaluating whether muscular fitness was consistently embedded from AHPEC’s stated rationale and aims, through the level descriptions and achievement standards to the detailed content elaborations, providing students with opportunities for developing muscular fitness within the curriculum.

### 2.3. Sampling Strategy

In the absence of a standardised list of muscular fitness terms, national government health agencies’ physical activity guidelines and muscular fitness intervention studies were used to compile a list of muscular fitness activity terms. Initially, the search strategy for national government health agencies’ physical activity guidelines was adapted from Parrish et al. [51] and included Google and Google Scholar searches (2010–2025) with site:org and site:gov limiters, screening of the first 15 pages, and targeted government and health organisation websites to identify relevant terms. Documents were included if they were published by a government or non-government organisation at the national level, represented the most current version available, and incorporated a clear statement outlining physical activity guidelines or recommendations for children and adolescents of school age. No language restrictions were applied. Documents were excluded if they were draft versions, superseded by another document, or took the form of news releases. Documents published in languages other than English were initially translated using digital translation. Translations were then reviewed by researchers with proficiency in the source language to verify accuracy and ensure conceptual equivalence with the original text. Any ambiguities were resolved through discussion.

In parallel, two researchers conducted a snowball search of Google Scholar [52], to identify muscular fitness activities described in intervention studies involving school-age children and adolescents. The initial search for intervention studies focused on school-based muscular fitness, using keywords such as “muscular fitness activities for children in schools” and “resistance training for students in schools”. This helped ensure the maximum number of different terms were being identified. Together, these processes generated a broad list of muscular fitness terms (activities) to guide data abstraction from the AHPEC.

### 2.4. Data Abstraction and Coding

National and international health agency documents were reviewed to identify references to muscular fitness. Documents were included regardless of original language and were translated into English for analysis (see Section 2.3). When multiple versions existed, the most recent guideline was used. Each document was coded for the depth of interpretation using the following codes: extrapolation, outline, description, or demonstration by example, and the number of muscular fitness activities explicitly identified. Activities were counted when specific examples (e.g., bodyweight, resistance exercises) were provided. Two researchers (K.T., A.S.) independently coded the agency documents, with disagreements resolved through discussion to ensure consistency.

The AHPEC Version 9.0 was accessed via the interactive online Australian Curriculum (Version 9.0) [5]. Data abstraction focused on identifying text and statements in the AHPEC from Foundation (Kindergarten) to Year 10 (F–10) that related to muscular fitness within the curriculum’s rationale and aim, the curriculum Level Descriptions and Achievement Standards, and the Movement and Physical Activity strand content elaborations. Each curriculum statement (rationale, aim, level description, achievement standard, content elaboration) was treated as a discrete unit of analysis. For the curriculum rationale and aim, references to muscular fitness were noted as either existing or absent. Curriculum level description and achievement standards per stage of schooling (Foundation Year = Kindergarten; Stage 1 [S1] = Years 1–2; Stage 2 [S2] = Years 3–4; Stage 3 [S3] = Years 5–6; Stage 4 [S4] = Years 7–8; Stage 5 [S5] = Years 9–10) were analysed for muscular fitness related statements and coded as ‘explicit’, ‘inferred’, or ‘not present’ to enable assessment of how muscular fitness was positioned in the high-level intentions of the curriculum and the extent to which these intentions were reflected in the detailed learning opportunities (see Table 1).

For curriculum content (i.e., content elaborations) in the ‘Movement and Physical Activity’ strand, data abstraction was conducted per stage of schooling. Firstly, identification of all content within the ‘Movement and Physical Activity’ strand and its sub-strands was conducted, as these represent potential teaching and learning opportunities available for integrating muscular fitness across the curriculum. From this initial step, the total number of content points and potentially relevant content points that could address muscular fitness were identified. References were coded according to pre-determined criteria (see Table 2), adapted from curriculum alignment methodologies [53]. Three researchers independently conducted data abstraction and coding, ensuring consistency and transparency in the classification process. Intercoder reliability was established through regular consensus meetings, during which discrepancies were discussed until full agreement was reached. Together, these procedures constituted a qualitative content analysis designed to investigate the representation of muscular fitness within the AHPEC.

### 2.5. Synthesis and Reporting of Results

Data abstraction and coding findings were synthesised to evaluate the extent to which and how the curriculum addressed the muscular fitness needs of children and adolescents of school age. Findings obtained from comparing the muscular fitness content elaborations and the AHPEC rationale and aims, curriculum level description and achievement standards were synthesised to provide an understanding of the extent to which these critical components of the curriculum aligned. Following the qualitative coding and synthesis, a quantitative coverage scoring system was applied to evaluate the comprehensiveness of muscular fitness content across curriculum stages.

#### 2.5.1. Quantitative Assessment of Muscular Fitness Coverage in Curriculum

To assess the extent to which muscular fitness is addressed across different stages of schooling, a quantitative scoring approach was applied. This method provided a systematic evaluation of the curriculum’s content related to muscular fitness, measuring the comprehensiveness of its coverage [47]. After identifying relevant (i.e., direct alignment) muscular fitness content for each educational stage (Foundation through Stage 5; see Section 2.4), a coverage score (CS) was calculated for each stage to quantify the proportion of muscular fitness-focused (directly aligned) content relative to the total available content points. The coverage score was determined using the following formula:

$$Coverage\;score\;(CS)=\frac{Number\;of\;muscular\;fitness\;directly\;aligned\;content\;points\;in\;curriculum}{Total\;number\;of\;content\;points}\times 100$$

The result of this calculation was a percentage indicating the extent of coverage for directly aligned muscular fitness content in each stage, from Foundation to Stage 5. To interpret these scores meaningfully, we applied a set of thresholds to categorise the levels of coverage as High (75–100%), Moderate (50–74%), Low (1–49%), or Absent (0%). This categorization provided clarity on whether each stage was substantially addressing muscular fitness or if gaps existed in the curriculum.

#### 2.5.2. Alignment of the Curriculum with Muscular Fitness

To understand the overall direct alignment of the curriculum with muscular fitness content across primary and secondary education, we calculated an aggregate coverage score for each school division. For primary education (F–6), the unweighted mean of coverage scores from Foundation Stage to Stage 3 was determined. For secondary education (Years 7–10), the mean of scores from Stage 4 and Stage 5 was used. An overall F–10 coverage score was then calculated as the unweighted mean across all stages, ensuring equal representation of each stage in the developmental pathway. The formulas for each division’s coverage are as follows:

$$Overall\;curriculum\;coverage\;primary=\frac{\sum Coverage\;scores\;for\;Foundation-Stage\;3}{Number\;of\;stages}$$

$$Overall\;curriculum\;coverage\;secondary=\frac{\sum Coverage\;scores\;for\;Stage\;4-Stage\;5}{Number\;of\;stages}$$

$$Overall\;curriculum\;coverage\;F–10=\frac{\sum Coverage\;scores\;for\;Foundation-Stage\;5}{Number\;of\;stages}$$

## 3. Results

The results of the search strategy for national physical activity guidelines are presented in Table 3. Thirty-two national and one international guideline (i.e., WHO) on physical activity were identified. Most (i.e., >50%) guidelines targeted children aged 5–17 years, although some extended to 4 years of age (in Germany, Greece, and the Netherlands) or 18–19 years of age (in Austria, India, Greece, Germany, Chile, Turkey, Singapore, United Kingdom). Most countries recommend ≥ 60 min of MVPA daily. Exceptions included Germany, which recommended ≥ 90 min, and India, which recommended 30–60 min. Chile specified 60–90 min per day. The majority of guidelines emphasised concurrent training with aerobic activity as the primary modality, supplemented by muscle- and bone-strengthening activities (MBSA) on at least three days per week. Variations included Spain and the United Kingdom (UK), which additionally recommended flexibility or movement skill activities, and Singapore and the UK, which highlighted “all forms” of physical activity. Frequency recommendations were consistently daily, except for Jamaica (five days per week) and Turkey guidelines, which limited MBSA to adolescents aged 12–18 years.

Analysis of national physical activity guidelines showed substantial variation in the depth of interpretation and the extent to which muscular fitness activities were identified (Table 4). Of these, 17 national guidelines (53.1%) contained no identifiable muscular fitness activities, while 15 (46.9%) provided at least one example. Across all guidelines, 88 muscular fitness activities were identified, with the number per guideline ranging from 0 to 17. Malaysia reported the highest number (17 activities, 19.3% of the total identified), followed by the USA (10 activities, 11.4%), the UK (8, 9.1%), Mexico (7, 8.0%), Qatar (7, 8.0%), and Australia (7, 8.0%). Other countries provided smaller numbers, including Germany (2, 2.3%), Peru (2, 2.3%), New Zealand (3, 3.4%), and Switzerland (4, 4.5%). In most cases where activities were identified, these were presented as descriptions or demonstrations by example, whereas over half of the guidelines required extrapolation to provide meaning without explicit examples. Overall, most agencies either provided no muscular fitness activities or listed only a limited number, with detailed demonstrations confined to a small subset of countries.

The national physical activity guidelines across 32 countries suggest a diverse range of activities for muscular fitness. The most common being climbing (11%), bodyweight resistance exercises (9%), jumping (9%), and lifting weights (8%), followed by gymnastics (6%), push-ups (6%), resistance bands (4%), resistance exercises (4%), jump rope (3%), hopping (3%), strength training (3%), sit-ups (3%), basketball (3%), and running (3%), while other recommended activities such as pushing, martial arts, monkey bars, playground equipment, tennis, pulling, tug-of-war, skipping (each 2%), as well as pull-ups, hill walking, sport, squats, star jumps, football, cycling, carrying, and yoga (each 1%) are also represented at lower percentages.

A total of 22 distinct muscular fitness activities were identified in the literature (Table 5). These were classified into six categories: (i) bodyweight and foundational training (6 activities: ballistic strength training [90], bodyweight strength exercises [91], callisthenics exercise group [92,93], Fundamental Integrative Training (FIT) [33], static strength training [upper body, lower body, core], and circuit training [93,94,95]); (ii) resistance-based training (4 activities: free-weight strength training [96], resistance training including weight machines, free weights, elastic bands, medicine balls, and plyometrics [91,97,98,99,100], resistance training machines [101], and traditional strength training [102]); (iii) power and explosive training (4 activities: plyometrics [97,100,102,103,104], power training [104], medicine ball training [104,105,106,107], and circuit strength training with plyometrics and strength exercises [108,109,110]); (iv) integrative and neuromuscular approaches (3 activities: fundamental integrative training [33], integrative neuromuscular training [111,112,113], and manual strength training [114]); (v) high-intensity and conditioning approaches (2 activities: high-intensity interval training [115,116,117] and CrossFit [118]); and (vi) novel or alternative modalities (4 activities: Bosu-based training [119,120,121], elastic tube strength training [96], suspension training [99], and trampolining [122,123,124]).

For the AHPEC rationale and aim, references related to muscular fitness were absent and therefore, no further analysis of these curriculum items was undertaken. Analysis of the curriculum level descriptions and achievement standards revealed a developmental shift in how muscular fitness is represented (see Supplementary File, Tables S1 and S2, pp. 3–5). In the early years (Kindergarten to Year 4), references to muscular fitness were consistently inferred, with muscular fitness suggested through descriptions of locomotor, object control, and movement performance, but never named directly. For Years 5–6, a divergence emerged: the curriculum level description made no reference to muscular fitness (i.e., not present), while the achievement standard explicitly introduced fitness as a concept linked to health and wellbeing. From Years 7–10, level descriptions continued to infer muscular fitness through performance and skill language, whereas achievement standards consistently made explicit references to fitness outcomes, encompassing muscular fitness. This pattern suggests that muscular fitness is implied throughout the early and middle years of learning but becomes explicitly recognised as a component of student achievement from Year 5 onwards.

Analysis of the curriculum content elaborations revealed differences in how muscular fitness is represented across the stages of learning (see Supplementary File, Tables S3 and S4, pp. 6–10). In the primary years, muscular fitness content was most often partially and peripherally aligned, with learning focused on locomotor skills, balance, object control, and movement challenges. A small number of directly aligned references appeared in Stage 2 and Stage 3, often through balance activities, gymnastics-like movements and plyometrics (i.e., jumping, landing and movement sequences), or links to the Australian 24-h Movement Guidelines, which reinforce the WHO and Australian Physical Activity Guidelines [126]. In contrast, the secondary years featured a relatively more explicit emphasis on directly aligned muscular fitness content, including fitness circuits, structured resistance-based activities, physiological monitoring, gym classes, high-intensity interval training (HIIT) sessions, and personal fitness planning. This progression indicates that overall muscular fitness is not a prominent or explicit component in primary schooling, becomes an explicit area of focus during secondary schooling, and remains a minor context.

The coverage scores across the stages of schooling showed that directly aligned muscular fitness content elaborations accounted for a small proportion of total content points. In the primary years the coverage scores were: Foundation, 0 of 18 points (0%—Absent) were directly aligned; Stage 1, 0 of 20 points (0%—Absent) were directly aligned; Stage 2, 2 of 33 points (6.1%—Low) were directly aligned; and in Stage 3, 3 of 33 points (9.1%—Low) were directly aligned. In the secondary years, 3 of 35 points (8.6%—Low) were directly aligned in Stage 4, and 3 of 34 points (8.8%—Low) were directly aligned in Stage 5. At no stage did directly aligned content account for more than 10% of the total content. The overall coverage scores for primary, secondary, and F–10 were 3.8%, 8.7%, and 5.4%, respectively, and were classified as ‘Low’.

## 4. Discussion

The present study was designed to determine the extent to which national physical activity guidelines recommend muscular fitness as an important adjunct to physical activity and to what extent AHPEC addresses the muscular fitness needs of students from F–10 as a priority, especially considering the plethora of benefits documented in research over the last 15 years [38,127]. Regarding the need for muscular fitness activities among school students, an analysis of national physical activity guidelines worldwide, including those from the WHO, confirms that children and adolescents across diverse populations require engagement in muscular fitness activities such as bodyweight and resistance-based exercises for optimal health. This strong global consensus on the central role of muscular fitness underscores the need to follow most (>95%) of the guidelines, which recommend concurrent training; daily moderate-to-vigorous activity combined with muscle- and bone-strengthening activities on at least three days per week. This position aligns with Australian Physical Activity Guidelines and reflects a broader public health policy emphasis on embedding muscular fitness as a foundation for healthy growth, bone strength, and lifelong participation in physical activity. For broader public health policies to be effective, translation into educational practice is essential; therefore, primary and secondary school HPE curricula must align explicitly with public health policy by including structured and adequate amounts of muscular fitness activities. Such alignment ensures that schools contribute directly to national health objectives while equipping students with the physical competence and habits necessary to sustain wellbeing throughout their lifespan.

The overarching result of the analysis shows that muscular fitness is inadequately and unevenly represented across the AHPEC. In the primary years, muscular fitness is rarely made explicit in curriculum-level descriptions and achievement standards, with most references coded as inferred through locomotor, balance, and movement proficiency, and in some instances, not presented at all. Furthermore, the current investigation found that references to muscular fitness are largely inferred in primary school curriculum-level descriptions and content elaborations (Foundation to Year 4). In contrast, in Year 5 and secondary school (i.e., Years 7–10), there is limited explicit reference. Hence, the curriculum stages fell within the Absent (0%) or Low coverage (1–49%) thresholds, with no stage reaching ‘Moderate’ or ‘High’ coverage. While this sequencing in the AHPEC reflects a simplistic and common pedagogical assumption in education, that children should first master fundamental movement skills before progressing to structured fitness training [128], the findings raise important concerns.

The analysis of content in the primary school years shows that, within the limited content that may relate to muscular fitness concepts, principles, or engagement, it is mainly partially or peripherally aligned. It is therefore not surprising that despite the availability of contemporary research to inform best curriculum practice [127], evidence shows that muscular fitness in primary-aged children has declined markedly over the past three decades [15,129], a trend now described as paediatric dynapenia. Furthermore, a major problem with the findings is that muscular fitness is also strongly correlated with fundamental movement skill proficiency [130,131], meaning delays in its development may undermine both skill acquisition and long-term participation. Moreover, contemporary research highlights that resistance training is not only safe and effective for children but also the gold-standard modality for enhancing strength, endurance, power, metabolic health, and even cardiorespiratory function [38]. Taken together, these findings suggest that the implicit treatment of muscular fitness in the primary curriculum does not align with contemporary evidence or health priorities.

With respect to the third research question, it was found that in the secondary years, both curriculum level descriptions and achievement standards do make explicit, however limited, reference to muscular fitness, often through targeted activities such as circuits, training practices, and personal fitness planning. This developmental progression with AHPEC implies that muscular fitness is not a priority in primary years content and becomes a context of consideration during adolescence. Despite the limited explicit focus in secondary school HPE, the aim and rationale of the HPE syllabus make no mention or infers muscular fitness. It seems possible that these findings may be due to a broader curriculum and education policy bias towards aerobic fitness, which continues to dominate health messaging in schools and community programmes. While aerobic activity remains important and is promoted in the WHO physical activity guidelines, the consistent privileging of cardiorespiratory outcomes risks marginalising muscular fitness [132], despite mounting evidence over the last twenty years that resistance training contributes uniquely to physical literacy, injury prevention, mental health, and long-term disease protection [127]. Curriculum and policy reform to align the curriculum with current physical activity guidelines and emerging international consensus that strength training is a central pillar of children’s and adolescents’ physical activity could provide teachers with clearer direction and confidence to implement age-appropriate muscular fitness-based activities. In this way, curriculum policy could play a decisive role in addressing the decline in muscular fitness among Australian children and promoting lifelong health trajectories.

Prior studies involving adolescents have noted a positive relationship between physical literacy, muscular fitness and participation in muscle-strengthening activities [133]. Systematic reviews also demonstrate that resistance training interventions improve not only physical strength, but also lead to significant improvements in the physical and affective domains of physical literacy [134]. While the curriculum emphasises the development of physical literacy, inadequate opportunities for students to build muscular fitness within HPE programmes risk undermining this aim. Neuromuscular performance, muscular strength, and endurance are critical foundations for the acquisition of fundamental movement skills and for fostering confidence, resilience, and sustained participation in physical activity [131]. If muscular fitness is not intentionally developed, students may encounter barriers to skill mastery, reduced motivation, and diminished capacity to engage in lifelong active living.

With respect to the fourth research question, it was observed that there is a lack of coherent and progressive muscular fitness promotion within the curriculum to combat the widespread effects of physical inactivity. The role of muscular fitness in reducing paediatric dynapenia, improving neuromuscular performance, and reducing activity-related injuries as well as adverse health events in students is not reinforced by the two general AHPEC framework aims of HPE; “*acquire, apply and evaluate movement skills, concepts and strategies to respond confidently, competently and creatively in various physical activity settings*” and to “*engage in and create opportunities for regular physical activity participation as individuals and for the communities to which they belong*” [5]. Accordingly, as the aim and rationale establish the conceptual scope and pedagogical direction of the HPE curriculum, it is imperative that they explicitly articulate the role of muscular fitness to ensure coherence between curricular intent, instructional focus, and the development of students’ functional health capacities, in alignment with the WHO and Australia’s physical activity guidelines which both emphasise the inclusion of muscular fitness as a key component of health-related physical activity.

The observed absence of explicit inclusion of muscular fitness in the aims and rationale, which delineates the conceptual boundaries of HPE, carries important implications for the alignment between AHPEC’s stated intentions and its treatment of muscular fitness. These concerns are compounded by longitudinal evidence indicating a marked decline in muscular fitness among primary-aged children over the past three decades [15,135], alongside robust associations with proficiency in fundamental movement skills [136]. Early childhood and the lower primary years are critical for developing these foundational skills, which, in turn, support lifelong participation in physical activity. The current curriculum’s implicit treatment of muscular fitness in the early years may limit teachers’ opportunities to deliberately target strength and muscular development, suggesting a misalignment between the AHPEC aims and the adequacy of muscular fitness content, despite the WHO and Australian physical activity guidelines recommending muscular fitness activities for children. Embedding explicit muscular fitness objectives earlier in the curriculum could strengthen foundational skills, align with health guidelines, and help reverse the decline in children’s muscular fitness.

Making muscular fitness more explicit in lower-year curriculum descriptions could help bridge this identified gap, particularly given that directly aligned muscular fitness content accounted for less than 10% of total content at every stage of schooling, and was largely absent in the early primary years. Addressing this gap could also support improvements in both movement skill proficiency and long-term health outcomes. Existing research provides evidence of a critical “window of opportunity” in middle childhood (i.e., age 6–11) for accelerated neuromuscular and motor coordination adaptations, resulting from heightened responsiveness to exercise and physical training [137]. Moreover, evidence from several experimental studies has established that children and adolescents who commence athletic training with limited muscular fitness strength are more likely to have compromised neuromuscular control, leading to poor biomechanics and, in turn, an increased risk of musculoskeletal injuries [138]. Within the HPE context, implementing evidence-based training approaches that progressively build muscular fitness is essential for promoting holistic health, physical competence, and long-term wellbeing [139]. Therefore, providing students with structured opportunities to develop muscular fitness is a critical component of quality PE, while fostering confidence and motivation to engage in lifelong physical activity.

The last question in this study sought to determine where muscular fitness could be best integrated into HPE. Developing muscular fitness through a staged progression aligns closely with the AHPEC need for sequenced learning and development, and is reinforced by international research [140]. In the early years (Foundation–Stage 1), play-based neuromuscular training and low-level plyometrics (e.g., hopping, animal walks, balance tasks) could provide a safe and effective foundation for developing coordination, stability, and force absorption, consistent with recommendations that resistance training can begin in childhood if developmentally appropriate [38,141]. By Stage 2–3, introducing bodyweight circuits, rope climbs, and structured movement challenges would explicitly build endurance and strength, aligning with research that highlights the role of muscular fitness in supporting fundamental movement skills and lifelong participation [140,142]. These activities could also be feasibly incorporated into existing curriculum tasks through simple additions such as partner-based resistance challenges, locomotor strength games, or brief circuit stations embedded within regular skill practice or warmups. In secondary school (Stages 4–5), students could transition to structured and progressive resistance training, including supervised use of free weights, plyometric drills, and personalised fitness planning approaches shown to be safe and beneficial for bone health, metabolic function, and injury prevention [7,29,38]. This proposed progression therefore bridges curriculum intent with evidence-based practice, ensuring muscular fitness is not only implied but systematically embedded across childhood and adolescence.

Based on the findings of this content analysis, further high-level curriculum revisions are recommended to further strengthen the role of muscular fitness within AHPEC. Explicit references to muscular fitness, muscular strength and endurance should be embedded earlier in the primary curriculum Level Descriptions, rather than remaining implicit within achievement standards. Achievement Standards, incorporating explicit and assessable indicators of muscular fitness in the early years, to assist teachers in monitoring development. Finally, curriculum framing should be aligned with contemporary physical activity guidelines and the growing evidence base demonstrating that resistance training is safe, effective, and essential for both children and adolescents.

A number of limitations need to be noted regarding the present study. While the involvement of an HPE curriculum designer and a curriculum policy expert as researchers in the current study strengthened the interpretive validity, it remains limited by its reliance on document-based content analysis. Such an approach is confined to analysing the intended and prescribed HPE curriculum and therefore reflects only the concepts and emphases formally included within it. As a result, it is unknown whether teachers supplement the prescribed HPE curriculum with additional muscular fitness content. A further limitation relates to the amount of instructional time allocated to HPE practical lessons, which varies substantially across countries and, in some contexts, is minimal. Limited curriculum time may restrict inclusion of prescribed opportunities for students to engage in muscular fitness activities, potentially constraining the real-world enactment of guideline recommendations within school settings. The present investigation may also have been limited by challenges in detecting national physical activity guidelines published in languages other than English during the search process, as well as by the absence of stand-alone national guidelines in some countries that instead adopt World Health Organization recommendations.

## 5. Conclusions

The lack of action in the HPE curriculum to adopt scientific approaches to improving muscular fitness, fundamental movement skills, and athletic motor skills competencies has recently led to continuous calls for action by leading international research in paediatric exercise science for muscular fitness training-type activities to be promoted for engagement by children and adolescents [29,38,127,143,144,145]. This study provides the necessary justification for calls to action, with the Australian context as the leading driver.

Indeed, the HPE syllabus does not consistently draw upon contemporary research conducted in school settings focused on paediatric exercise science and muscular fitness. Furthermore, the curriculum appears to neglect the explicit promotion of muscular fitness in the primary years. As outlined earlier, this is anomalous given the strong evidence that muscular fitness is fundamental to children’s physical, cognitive, and social development in the 21st century. Given the absence of explicit opportunities for children and adolescents, there may be value in exploring adjustments to address this gap in the HPE curriculum, helping align it with contemporary research and further supporting learners’ holistic development. Such an approach would align curriculum design with contemporary evidence and facilitate schools’ more effective contribution to addressing pressing public health concerns, including childhood obesity, mental health, and long-term physical literacy [37]. Positioning muscular fitness as a core curricular priority ensures that education contributes directly to the promotion of functional longevity, resilience, and quality of life across the lifespan [23]. The current findings also highlight an important opportunity for future curriculum refinement to better align with international guidelines on muscular fitness for children and adolescents.

## Figures and Tables

**Table 1:** Coding key for muscular fitness embedded in stage-level descriptions and achievement standards.

Code Category	Operational Definition	Criteria for Inclusion	Example from Curriculum * Text
**Explicit**	Direct reference to muscular fitness or closely related constructs.	Curriculum text names muscular fitness or synonymous terms (i.e., muscular strength, endurance, power, resistance training, strength training, improving fitness)	“Students propose and evaluate strategies designed to achieve personal health, fitness and wellbeing outcomes.”
**Inferred**	Indirect reference to muscular fitness.	Curriculum text does not use explicit terms but implies muscular function through references to movement performance, control, stability, force production, or fitness practices.	“They practice techniques that can be used to enhance their own and others’ performances.”
**Not Present**	No reference to muscular fitness.	Curriculum text contains no explicit or inferred mention of muscular fitness.	“Students work collaboratively to solve movement challenges.”

Note: *: Australian Health and Physical Education Curriculum.

**Table 2:** Coding key for muscular fitness embedded in content elaborations.

Code Category	Operational Definition	Example from Curriculum Text *
**Direct Alignment**	Content that explicitly encompasses muscular fitness concepts, principles, or modalities. This includes learning experiences that intentionally develop muscular strength, power, endurance, or neuromuscular control through activities such as resistance training, plyometrics, circuit training, or bodyweight exercises.	“Researching and participating in new activities to explore how they can enhance health, fitness and wellbeing, such as yoga, mindfulness meditation, gym classes, HIIT sessions.”
**Partial Alignment**	Content in which there may be learning opportunities to include muscular fitness principles, concept development; however, they may occur indirectly or as a secondary emphasis. In addition, muscular fitness may be developed through activities that inherently require strength, power, or control, such as gymnastics, climbing, or apparatus work, even though the explicit learning intention focuses on skill execution, coordination, or movement quality rather than muscular fitness as a primary focus.	“Performing a range of movements and analysing technique based on understanding of effort in relation to take-off, body position and landing.”
**Peripheral Alignment**	Content that relates to general physical health, wellbeing, or participation, without explicit or implicit reference to muscular fitness, but may support it.	“Participating in a range of physical activities and investigating opportunities to incorporate these into lunchtime activities.”
**No Alignment**	The descriptor has no conceptual link to muscular fitness.	“Demonstrating negotiation skills when dealing with conflicts or disagreements in movement situations.”

Note: *: Australian Health and Physical Education Curriculum.

**Table 3:** Summary of national physical activity guidelines for primary to high school children by country.

Country	Year of Release	Age Group (yrs)	Time (min)	Intensity	Frequency	Concurrent Training	
						Primary Activity Modality	Secondary Activity Modality (Weekly Frequency)
Argentina [54]	2021	5–17	≥60	MV	Daily	Aerobic	MBSA (≥3)
Australia [55,56]	2019	5–17	≥60	MV	Daily	Aerobic	MBSA (≥3)
Austria [57]	2020	6–18	≥60	MV	Daily	Aerobic	MBSA (≥3)
Brazil [58]	2021	6–17	≥60	MV	Daily	AF *	MBSA (≥3)
Canada [59]	2016	5–17	≥60	MV	Daily	Aerobic	MBSA (≥3)
Chile [60]	2021	5–19	≥60–90	MV	Daily	Aerobic	Absent
China [61,62]	2021	6–17	≥60	MV	Daily	Aerobic	MBSA (≥3)
Colombia [63]	2021	5–17	≥60	MV	Daily	Aerobic	MBSA (≥3)
Finland [64]	2021	7–17	≥60	MV	Daily	Aerobic	MBSA (≥3)
France [65]	2019	6–17	≥60	MV	Daily	Aerobic	Absent
Germany [66]	2017	4–18	≥90	MV	Daily	Aerobic	MBSA (≥3)
Greece [67,68]	2017	4–18	-	MV	Daily	Aerobic	-
India [69]	2019	5–18	≥30–60	MV	Daily	Aerobic	MBSA (≥3)
Italy [70]	2021	5–17	≥60	MV	Daily	Aerobic	MBSA (≥3)
Jamaica [71]	2018	6–17	≥60	MV	5 days/week	Aerobic	MBSA (≥3)
Japan [72]	2023	5–17	≥60	MV	Daily	Aerobic	MBSA (≥3)
Kenya [73]	2018	5–17	≥60	MV	Daily	Aerobic	MBSA (≥3)
Malaysia [74]	2017	5–17	≥60	MV	Daily	Aerobic	MBSA (≥3)
Mexico [75]	2023	5–17	≥60	MV	Daily	Aerobic	MBSA (≥3)
The Netherlands [76]	2017	4–18	≥60	MV	Daily	Aerobic	MBSA (≥3)
New Zealand [77]	2025	5–17	≥60	MV	Daily	Aerobic	MBSA (≥3)
Paraguay [78]	2014	5–17	≥60	MV	Daily	Aerobic	MBSA (≥3)
Peru [79]	2025	6–17	≥60	MV	Daily	Aerobic	MBSA (≥3)
Qatar [80]	2021	5–17	≥60	MV	Daily	Aerobic	MBSA (≥3)
Saudi Arabia [81]	2021	6–17	≥60	MV	Daily	Aerobic	MBSA (≥3)
Singapore [82]	2021	7–18	≥60	MV	Daily	AF *	MBSA (≥3)
Spain [83]	2022	5–17	≥60	MV	Daily	Aerobic	MBSA, flexibility (≥3)
Switzerland [84]	2022	5–17	≥60	MV	Daily	Aerobic	MBSA (≥3)
Turkey [85]	2014	5–18	≥60	MV	Daily	Aerobic	MBSA (≥3) for 12–18-year-olds only
USA [86]	2018	6–17	≥60	MV	Daily	Aerobic	MBSA (≥3)
Uruguay [87]	2019	5–17	≥60	MV	Daily	Aerobic	MBSA (≥3)
United Kingdom [88]	2019	5–18	≥60	MV	Daily	AF *	MBSA and activities to develop movement skills across the week
WHO [89]	2020	5–17	≥60	MV	Daily	Aerobic	MBSA (≥3)

Note: MV: moderate-vigorous; MBSA: muscle and bone strengthening activities; AF: All forms; *: any form of physical activity.

**Table 4:** Analysis of national physical activity guidelines: depth of interpretation and identification of muscular fitness activities.

Origin of National Government Health Agencies	Depth of Interpretation and Demonstration of Muscular Fitness Activities				Number of Muscular Fitness Activities Identified
	Extrapolation	Demonstration by Example	Outline	Description	
Argentina [54]	✓				0
Australia [55,56]		✓	✓		7
Austria [57]	✓				0
Brazil [58]		✓		✓	4
Canada [59]	✓				0
Chile [60]	✓				0
China [61,62]	✓				0
Colombia [63]	✓				0
Finland [64]	✓				0
France [65]	✓				0
Germany [66]		✓		✓	2
Greece [67]					0
India [69]	✓				0
Italy [70]	✓				0
Jamaica [71]	✓				0
Japan [72]	✓	✓			1
Kenya [73]	✓				0
Malaysia [74]		✓		✓	17
Mexico [75]		✓	✓		7
The Netherlands [76]		✓		✓	6
New Zealand [77]	✓	✓			3
Paraguay [78]	✓				0
Peru [79]	✓	✓			2
Qatar [80]		✓		✓	7
Saudi Arabia [81]	✓				0
Singapore [82]		✓		✓	4
Spain [83]		✓		✓	6
Switzerland [84]	✓	✓			4
Turkey [85]	✓				0
USA [86]		✓		✓	10
Uruguay [87]	✓				0
United Kingdom [88]	✓		✓		8
WHO [89]	✓				0

**Table 5:** Screening of muscular fitness activities identified in the relevant literature.

Category	Activities Identified	References
**Bodyweight and Foundational Training**	Ballistic strength training; Bodyweight strength exercises; Callisthenics exercise group; Fundamental integrative training; Static strength training (upper body, lower body, core); Circuit training	[33,90,91,92,93,94,95,108]
**Resistance-Based Training**	Free-weight strength training; Resistance training (machines, free weights, elastic bands, medicine balls, plyometrics); Resistance training machines; Traditional strength training	[91,96,97,98,99,100,101,102,104,105,106,107,125]
**Power and Explosive Training**	Plyometrics; Power training; Medicine ball training; Circuit strength training with plyometrics and strength exercises	[97,100,102,103,104,108,109,110]
**Integrative and Neuromuscular Approaches**	Fundamental integrative training; Integrative neuromuscular training; Manual strength training	[33,111,112,113,114]
**High-Intensity and Conditioning Approaches**	High-intensity interval training; CrossFit	[115,116,117,118]
**Novel or Alternative Modalities**	Bosu-based training; Elastic tube strength training; Suspension training; Trampolining	[96,99,119,120,121,122,123,124]

## Data Availability

The original contributions presented in this study are included in the article/Supplementary Materials. Further inquiries can be directed to the corresponding author.

## Supplementary Materials

The following supporting information can be downloaded at: https://www.mdpi.com/article/10.3390/jfmk11010040/s1, Table S1: Reference to muscular fitness in primary school curriculum level description and achievement standards; Table S2: Reference to muscular fitness in secondary school curriculum level description and achievement standards; Table S3: Muscular fitness specific and related items in the primary school curriculum content; Table S4: Muscular fitness specific and related items in the secondary school curriculum content.

## Abbreviations

The following abbreviations are used in this manuscript:

AHPEC	Australian Health Physical Education Curriculum
MVPA	Moderate-vigorous physical activity
HPE	Health and Physical Education
