# Supplementary material for: The Health and Physical Education Curriculum: Does It Address Muscular Fitness?
Source: J Funct Morphol Kinesiol. 2026 Jan 18;11(1):40. doi: 10.3390/jfmk11010040 (PMC12821546; doi:10.3390/jfmk11010040)
Supplement: Supplementary file 1 [file jfmk-11-00040-s001.zip › jfmk-4016621-supplementary.pdf]

Supplementary appendix

The Health and Physical Education Curriculum: Does it Address Muscular Fitness?

Contents

Supplementary material 1 – Table S1. Reference to muscular fitness in primary school curriculum level description and achievement standards 3

Supplementary material 2 – Table S2. Reference to muscular fitness in secondary school curriculum level description and achievement standards 5

Supplementary material 3 – Table S3. Muscular fitness specific and related items in the primary school curriculum content 6

Supplementary material 4 – Table S4. Muscular fitness specific and related items in the secondary school curriculum content 9

Supplementary material 1 – Table S1. Reference to muscular fitness in primary school curriculum Level Description and Achievement Standards

Year Band	Curriculum Component	Curriculum Text (excerpt)	Code (Explicit/ Inferred/ Not Present)	Reason for Coding
**Foundation**	Band Level Description	"Students practise and develop locomotor and non-locomotor skills, including balancing, running and jumping, and object control skills, including rolling, propelling, bouncing, throwing, catching and kicking a range of different objects." and "Through participation in active play, small group games and minor games..."	Inferred	Activities such as balancing, running, and jumping require muscular strength and endurance, but muscular fitness is not explicitly stated.
	Band Achievement Standards	"Students apply fundamental movement skills to manipulate objects and space in a range of movement situations. Students identify the benefits of being physically active…"	Inferred	Manipulating objects and space requires muscular strength and coordination, though muscular fitness is not named directly.
**Years 1-2**	Band Level Description	"Through the continued development of fundamental skills, students participate in a range of different physical activities… They refine and extend skills… including rolling, leaping, skipping, galloping and dodging, and object control skills such as striking and kicking."	Inferred	Locomotor and object control skills suggest muscular engagement and development, but muscular fitness is implied rather than named.
	Band Achievement Standards	"Students apply fundamental movement skills in different movement situations and explain how they move with objects and in space effectively. They describe factors that make physical activity beneficial."	Inferred	Effective movement with objects implies muscular control, but no explicit mention of muscular fitness.

Reference to Muscular fitness in primary school curriculum level description and achievement standards - continued

Year Band	Curriculum Component	Curriculum Text (excerpt)	Code (Explicit/ Inferred/ Not Present)	Reason for Coding
**Years 3-4**	Band Level Description	"Students develop greater proficiency across the range of fundamental movement skills by building on previous learning. They practise and refine the skills introduced in the early years and transfer them to unfamiliar movement situations."	Inferred	Proficiency in movement skills depends on muscular strength and endurance; however, no direct terminology for muscular fitness is used.
	Band Achievement Standards	"Students apply fundamental movement skills and demonstrate movement concepts across a range of situations. They adapt movement strategies to enhance movement outcomes."	Inferred	Enhancing movement outcomes implies muscular contributions, though muscular fitness is not explicitly stated.
**Years 5-6**	Band Level Description	"Students explore ways they can actively participate in a range of physical activity settings and contribute to building a healthier community."	Not present	Focuses on participation and community health without explicit or implied reference to muscular fitness.
	Band Achievement Standards	"Students refine and modify movement skills and apply movement concepts across a range of situations… Students propose strategies to promote physical activity participation that enhance health, fitness and wellbeing."	Explicit	The reference to enhancing fitness (as part of health, fitness, and wellbeing) directly encompasses muscular fitness.

Supplementary material 2 – Table S2. Reference to muscular fitness in secondary school curriculum level description and achievement standards

Year Band	Curriculum Component	Curriculum Text (excerpt)	Code (Explicit/ Inferred/ Not Present)	Reason for Coding
**Years 7-8**	Band Level Description	"Through opportunities for skill development in a variety of movement forms that enhance performance and competence…" and "They practise techniques that can be used to enhance their own and others' performances."	Inferred	References to enhancing movement performance and practising techniques can imply muscular fitness, but it is not explicitly identified.
	Band Achievement Standards	"Students apply and transfer movement skills and movement concepts…" and "Students propose and evaluate strategies designed to achieve personal health, fitness and wellbeing outcomes."	Explicit	Explicit mention of achieving fitness outcomes includes muscular fitness.
**Years 9-10**	Band Level Description	"Students practise and refine more specialised movement skills and complex movement strategies and concepts in different movement environments. They apply movement concepts and strategies to evaluate and refine their own and others' movement performances." and "They adapt and improvise their movements to respond to different movement situations, stimuli and challenges."	Inferred	Refining specialised and complex movement skills implies strength and muscular control, but muscular fitness is not directly stated.
	Band Achievement Standards	"Students evaluate and refine their own and others' movement skills and performances…" and "Students propose and evaluate community-based physical activity interventions designed to improve the health, fitness and wellbeing of themselves and others."	Explicit	Explicit reference to improving fitness outcomes encompasses muscular fitness.

Supplementary material 3 – Table S3. Muscular fitness specific and related items in the primary school curriculum content

Target	Direct alignment	Partial alignment	Peripheral alignment
**Primary, Foundation**		• "…combining fine and gross motor skills in increasingly complex patterns…"<br>• "…applying different locomotor skills to move from one point to another.."<br>• "…sending, controlling and receiving objects at different levels and in different ways…"<br>• "…applying fundamental movement skills purpose and enjoyment in natural environments…"	• "…demonstrating how to transfer weight from one part of the body to another…"
**Primary, Stage 1 (Years 1–2)**		• "…performing locomotor movements using different body parts to travel in different directions…"<br>• "…demonstrating changes in speed, direction and level as they use locomotor and non-locomotor skills in sequences…"<br>• "…performing fundamental movement skills involving controlling objects with equipment and different parts of the body…"	• "…selecting and implementing different movement skills to be successful in a range of games…"<br>• "…demonstrating balances and describing what helps to maintain stable positions…"<br>• "…comparing the characteristics and benefits of physical activities that can take place in an outdoor setting to those that take place inside…"<br>• "…describing and demonstrating how to include others in physical activities…"

Muscular fitness specific and related items in the primary school curriculum content - continued

Target	Direct alignment	Partial alignment	Peripheral alignment
**Primary, Stage 2 (Years 3–4)**	• "…exploring physical activity and screen-usage time recommendations in the Australian 24-Hour Movement Guidelines for Children and Young People and proposing how they can meet these recommendations…"<br>• "…performing routines incorporating different jumping, landing and balancing techniques, and connecting movements to create a movement sequence…"	• "…performing activities where locomotor and object control skills are combined to complete a movement…"<br>• "…performing fundamental movement skills to demonstrate weight transference…"<br>• "…transferring and applying fundamental movement skills to solve movement challenges…"<br>• "…developing questions and seeking and trialling answers with others as a strategy for solving movement challenges, such as partner or group balance challenges…"	• "…exploring and practising different techniques to propel objects…"<br>• "…demonstrating acceleration, deceleration and changing direction of movement in minor games, and rhythmic and expressive activities…"<br>• "…participating in physical activities in natural settings…"<br>• "…participating in physical activities they can do at home…"<br>• "…exploring different ways of manipulating space to receive passes, maintain possession, or increase or decrease scoring opportunities in invasion, net/court, striking and fielding, and target games…"<br>• "…participating in physical activities in natural settings…"<br>• "…participating in a range of physical activities and investigating opportunities to incorporate these into lunchtime activities…"<br>• "…examining the benefits of regular physical activity, including the influence on sleep, concentration and wellbeing…"

Muscular fitness specific and related items in the primary school curriculum content - continued

Target	Direct alignment	Partial alignment	Peripheral alignment
**Primary, Stage 3 (Years 5–6)**	• "Researching the Australian 24-Hour Movement Guidelines for Children and Young People, comparing their daily habits of physical activity to the recommendations and proposing strategies for enhancing or maintaining their levels of activity."<br>• "Creating and participating in an activity circuit they could replicate at home that is designed to improve health-related fitness."<br>• "…participating in and designing physical activity opportunities that support their health and fitness goals…"	• "…performing activities that involve a transition from one skill to another, such as from dribbling to shooting or from leaping to balancing, in changing movement situations…"<br>• "…demonstrating an understanding of how to adjust the force and speed of an object to improve accuracy and control…"<br>• "…working with a partner to explore pushing and pulling movements and how these can be manipulated to generate and perform counterbalances…"<br>• "…performing activities of different intensities and measuring breathing rate, heart rate and other body responses to categorise activities into low, moderate and high-intensity…"<br>• "…composing and performing a range of static and dynamic balances on different body parts, rotating and pivoting to change direction of movement…"	• "…combining surface propulsion and underwater skills in an aquatic environment…"<br>• "…analysing how access to natural environments and the outdoors can influence participation in physical activities, and enhance health and wellbeing."<br>• "…examining the benefits of physical activity for social health and mental wellbeing, and researching options for participating in physical activities in the local area…"<br>• "…investigating the resources needed and steps required to set up a lunchtime sports competition, activity circuit or playground games aimed at increasing levels of physical activity among students and staff."

Supplementary material 4 – Table S4. Muscular fitness specific and related items in the secondary school curriculum content

Target	Direct alignment	Partial alignment	Peripheral alignment
**Secondary, Stage 4 (Years 7–8)**	• "…using digital tools to design personal physical activity programs that support regular participation and meet their individual health, wellbeing and fitness goals."<br>• "…researching and participating in new activities to explore how they can enhance health, fitness and wellbeing, such as yoga, mindfulness meditation, gym classes, HIIT sessions…"<br>• "…comparing their current physical activity levels, amount of sleep and sedentary activity time with Australia's 24-Hour Movement Guidelines for Children and Young People and suggesting strategies for themselves and others to meet these recommendations."	• "…designing and monitoring a personal physical activity plan that proposes realistic strategies for increasing physical activity levels and reducing sedentary behaviours…"<br>• "…performing a range of movements and analysing technique based on understanding of effort in relation to take-off, body position and landing…"	• "..creating, performing and appraising rhythmic movement sequences that demonstrate variations in flow of movements, use of space and relationships to other performers…"<br>• "…explaining and justifying the movement strategies selected in response to movement challenges …. when participating in outdoor or nature-based activities such as rope courses…"

Muscular fitness specific and related items in the secondary school curriculum content - continued

Target	Direct alignment	Partial alignment	Peripheral alignment
**Secondary**<br>**Stage 5**<br>**(Years 9–10)**	• "…performing a range of activities designed to improve fitness and analysing how the activities improve individual components of fitness."<br>• "…using digital tools to design, implement and monitor a personal fitness plan that includes a timeframe, goals and a variety of specific activities that meet the needs of different people"<br>• "…investigating target training heart-rate zones for a range of different people, how these zones can be measured and how they relate to health, wellbeing and fitness levels"	• "participating in a range of physical activities and evaluating individual responses such as heart rate, breathing rate, ability to talk and recovery rate"<br>• "setting realistic physical activity goals, and designing, implementing and evaluating a personalised program to incorporate regular physical activity into their weekly routines"<br>• "justifying the selection of physical activities included in a personalised plan linked to physical activity goals and wellbeing outcomes they wish to improve or maintain"<br>• "speculating on possible outcomes of innovative solutions to movement challenges…. rope climbing"	• "experimenting with the manipulation of force and speed applied to an object"<br>• "analysing a range of ways the body can absorb force..."<br>• "investigating community-based campaigns to promote physical activity participation and determining key elements of success that could be replicated in a school-based campaign"
